# Why do People Help Each Other? Motivations of Volunteers Who Assisted Persons with Disabilities During World Youth Day

**DOI:** 10.1007/s10943-018-0625-z

**Published:** 2018-04-20

**Authors:** Edyta Janus, Anna Misiorek

**Affiliations:** Department of Occupational Therapy, University of Physical Education in Cracow, Al. Jana Pawła II 78, 31-571 Kraków, Poland

**Keywords:** Motivation, Helping others, Volunteerism, World Youth Day

## Abstract

A growing number of researchers attempt to identify the reasons why some people become volunteers. An analysis of the motives of people who helped persons with disabilities for free during World Youth Day, a renowned religious event, may contribute to this discussion. The aim of this article is to present the results of a survey encompassing 51 volunteers who assisted persons with disabilities during the 31st World Youth Day, which was held in Poland in 2016.

## Introduction

In July 2016, the World Youth Day (WYD) was held in Krakow, Poland. This cyclic meeting of youth was initiated by Pope John Paul II in 1985. As in the previous years, the 31st edition of the event was attended by people from different cultures and nationalities. These people spent time together not only praying, but also integrating and making friends.

Volunteers played an important role during the event. According to the basic definitions, “volunteerism” denotes helping others without expecting financial rewards. It is a type of activity that aims to improve the well-being of other people (Mowen and Sujan 2005). Other definitions describe volunteerism as volitional and planned helping behaviors that improve the well-being of the recipients of these behaviors. Volunteering is an important social behavior, needed to sustain civil society (Cnaan and Park 2015; Smith et al. 2016). Voluntarism usually occurs in the context of an organization, which means that it is performed under the auspices of particular entities (Finkelstien 2009). The above-mentioned definitions correspond to Polish regulations, according to which a volunteer is a person who volitionally offers to provide services for the beneficiaries willingly and free of charge. To become a volunteer during World Youth Day, a person was required to meet a few conditions, such as: be of legal age, complete a form with personal data, present a letter of recommendation from his or her parish priest, prior, or coordinator of his or her apostolic movement/association, and pay the corresponding fee (Volunteering Regulations of World Youth Day 2016). Additionally, these persons were obligated to take part in training appropriate to the type of volunteer work. One of the volunteer groups comprised people who volunteered to help people with disabilities wanting to participate in WYD, but who needed support due to their own limitations. An organization commissioned by Krakow to actively recruit and train volunteers in how to support persons with disabilities during WYD was the KLIKA Catholic Association for Persons With Disabilities and Their Friends [Polish: Katolickie Stowarzyszenie Osób Niepełnosprawnych i Ich Przyjaciół “KLIKA”]. The association has cooperated with the Dominican Monastery in Krakow for 45 years, providing support for persons with disabilities in their daily lives and enabling them to take part in education and recreation, and cultivate their interests.

Displaying helping behaviors toward persons with disabilities may result from various motivations guiding the people who are offering the help. As the subject of volunteerism has been gaining popularity for over a decade, numerous theoretical models explaining this phenomenon have appeared (Omoto and Snyder 1995; Penner and Finkelstein 1998). The notion of “motivation,” which forms an important part of these theoretical models, is crucial to understanding why individuals participate in voluntary activities. It is these motivations that this article attempts to analyze. In the context of WYD, the question about the significance of factors that are directly related to and resulting from the faith among these motivations seems justified.

All quotes presented in this paper have been translated from Polish for the purpose of this article.

## Material

The study encompassed 75 volunteers who supported persons with disabilities during World Youth Day. The sample was deliberately selected from among the members of the KLIKA Association who volunteered to provide care to persons with disabilities during WYD in Krakow. The volunteers responded to posters that had been hung in parishes, universities, and chaplaincies in Krakow, or they learned about the opportunity to be a volunteer assisting persons with disabilities during WYD on the association’s website. A total of 130 persons applied. Of these, 75 actually provided support for persons with disabilities. The KLIKA Association gave each person tasks to carry out, which mostly involved helping a particular person with disability arrive and participate in WYD, and securing food services and efficient organization of particular events. All 75 volunteers were asked to complete the questionnaire; of these, 51 (*N* = 51) agreed to do so. All participants provided written informed consent. All surveyed persons were Polish.

## Method

The study used a survey prepared by the authors of the study, which comprised 11 questions and respondent’s particulars. Five of the questions were open-ended and asked about the motives for undertaking voluntary service for persons with disabilities during WYD, previous experience with volunteerism, personal traits that make it easier to be a volunteer, etc. Three questions were semi-open and asked about factors pertaining to volunteer work that the respondents considered as rewarding and the possible hardships they encountered. The remaining questions were close-ended and asked if the respondents considered themselves as believers, etc. At the end of the questionnaire, the respondents were asked to provide information about their age, gender, place of residence, the duration of their volunteer work during WYD, and any disabilities they had.

The data were collected in Krakow in July 2016. The respondents completed a paper version of the survey. Details of demographic characteristics are listed in Table 1.

**Table 1:** Demographics data. *Source*: Private findings

Category	*N*	%
Sex		
Female	36	70.6
Male	15	29.4
Age (in years)		
18–25	29	57.0
26–35	15	29.4
34–40	2	3.9
41–47	5	9.7
Type of disability		
Without disability	48	94.1
Minor disability	1	1.9
Moderate disability	2	4.0

## Results

The survey began with an open-ended question about why the respondents had volunteered to assist persons with disabilities during World Youth Day in Krakow. The answers were analyzed with respect to two categories: altruistic motives and social motives. Altruistic motives emphasize the importance of caring for others. Social motives refer to interpersonal relationships (Monga 2006). An analysis of the obtained answers allows for the conclusion that altruistic motives, which are expressed through statements such as, “I want to help others, do something for others,” “I want to help less able people and make it possible for them to participate in World Youth Day,” and “I have experience in working with persons with disabilities and I want to share it,” were the dominant motives behind volunteering to assist persons with disabilities. The above-mentioned altruistic motives can be divided into exogenous and endogenous (Karyłowski 1982). Endogenous altruistic motives are in effect when a person is motivated by an internal reward in the form of maintaining or improving their self-esteem (a motivation factor in the form of anticipation of desirable changes or avoiding undesirable changes in one’s self-image). The exogenous motives are in effect when a person is oriented toward other people and is driven by the desire to improve the other person’s situation or prevent it from worsening (a motivation factor in the form of anticipation of desirable changes, i.e., the improvement in a situation or avoidance of an undesirable change in other people’s situations). The responses allow for the conclusion that the exogenous motives were slightly dominant among the respondents, as shown by the quoted reasons, which focus on enabling individuals with disabilities to participate in a religious event and helping them without consideration for one’s own profits. As far as the social motives are concerned, which were significantly less frequent than the altruistic motives, statements such as “I do this because I was encouraged by friends who are volunteers,” “I want to learn the joy of life from others,” or “I have been acting in the community for years; it is only natural that I get involved in this kinds of activities” were dominant. Reasons that can be linked directly to the religiosity are also noteworthy, albeit few in number. It is worth quoting some of them: “I want to show the importance of Christian values,” “I want to strengthen my faith,” and “I want to do something more than practicing the faith in a church.”

The next question asked if the respondents had any prior experiences with volunteerism. The vast majority of the respondents (90%, 46 persons) indicated that they were experienced volunteers. The share of respondents who declared that volunteerism during WYD was a new experience for them and they had never provided that form of assistance equaled 10% (5 persons). Among the persons who had experience in volunteerism, those who had assisted others regularly for months and years were dominant and constituted 32.6% (15 persons). Once averaged, the results indicate that these persons had been volunteers for around 10 years. The smallest number of respondents (13%, 6 persons) declared that they had been volunteers six or more times. Table 2 presents the detailed results.

**Table 2:** Times engaged in volunteer work. *Source*: Private findings

Times engaged in volunteerism	*N* (46)	%
1 time	11	24
2–5 times	14	30.4
6 times or more	6	13
Regularly for months/years	15	32.6

According to these data, it can be concluded that the majority of the volunteers had a “history of helping,” and that helping others was part of their daily lives. The most frequently mentioned initiatives in which the volunteers had been involved are as follows: working for the KLIKA Catholic Association for Persons with Disabilities (which supports the development of persons with disabilities in the most important spheres of life: physical, social, and professional), participation in the Noble Box [Polish: Szlachetna Paczka] (an initiative that involves raising funds and satisfying the material needs of persons in a difficult life situation), volunteerism during cultural events, working for Caritas (a charitable institution of the Polish Episcopal Conference, which provides short- and long-term material, psychological, legal, and financial help for children, and unemployed, homeless, sick, and elderly persons), and volunteer work for the elderly and children.

The next question asked if the respondents had ever encountered any obstacles that might have affected their decision to engage in volunteerism during WYD; 64.7% of the respondents (33 persons) declared that they did not encounter any difficulties. Participation in the event involved complications for 35.3% (18 persons) of the respondents. The following difficulties can be considered as the most significant: difficulties with reorganizing professional duties connected with absence at work during WYD, and the lack of understanding from one’s family manifested as a lack of acceptance of the fact that a given person intended to work as a volunteer. The respondents also indicated the fear of a terrorist attack, which made them uncertain about whether to take part in the event. Despite the above-mentioned difficulties, the respondents made the effort to overcome them in order to be able to fully participate in WYD.

The next issue raised in the questionnaire focused on what the volunteers considered to be the most rewarding in volunteerism. This was a semi-open question. The majority of the respondents considered “doing good” as an excellent reward for volunteerism during WYD. “Carrying out God’s will” placed second. Many respondents also indicated “satisfaction from participating in and contributing to a religious event in the form of WYD.” None of the surveyed persons indicated that praise was rewarding for them. Table 3 presents detailed results. The respondents could choose no more than three factors.

**Table 3:** Rewarding factors in volunteering. *Source*: Private findings

Name of factor	Rank	Number of responses
Doing good	1	37
Carrying out God’s will	2	24
Satisfaction from participating in and contributing to a religious event in the form of WYD	3	22
Deepening reflection on the purpose and sense of life	4	14
Establishing new interpersonal relations	6	14
The feeling of being needed	7	8
Proving oneself in a new situation	8	7
Perfecting one’s skills (linguistic, organizational)	9	6
The awareness that there will be someone to help me when I am in need	10	5
Other	11	2
Receiving praise	12	0

The rewarding factors described in the table correspond with the answers for the question about the motivation to become a volunteer. The respondents’ answers indicate that the exogenous motives dominated. “Doing good” as a rewarding factor naturally implies the desire to support others. Helping others may result from the internalized principles of the faith. The respondents referred to themselves as believers. The answer “Yes, I am a believer” was given by 98% of respondents. Only one person gave the answer “No, I am not a believer.” The results concerning identification with a particular Christian tradition are similar. Ninety-eight percent of respondents identified themselves with Catholicism. One person declared that they did not identify with any of the listed Christian traditions.

In the next part of the questionnaire, the surveyed persons were asked to describe how they would encourage other persons to become volunteers. The answers were analyzed with respect to altruistic motives and social motives, just like the answers to the question about the reasons for participating in WYD as volunteers who supported persons with disabilities. Recommending volunteerism to others, the respondents emphasized, first and foremost, that helping others brings benefits not only to the recipient of the help, but also to the helper. The statements corresponding with the altruistic motives for helping others reflect this well: “Do something that will make you and the others happy,” “Do something for others and you will receive even more,” “Helping others allows you to discover yourself and your humanity,” “By helping others, we fight our own egoism,” “Helping others gives my life meaning,” and “It is worth helping and being needed.” The answer shows that the altruistic exogenous motives were the dominant ones. Social motives were indicated relatively rarely. As an example, we can quote the following statements: “When you work as a volunteer, you experience life in a community,” “Volunteerism is a beautiful adventure with another human,” “Volunteerism is a way of meeting new people and gaining new experiences,” and “Volunteerism is an extraordinary opportunity to meet new people.” The answers directly referring to religion were of marginal significance among the obtained replies. Nevertheless, it is worth quoting them: “Volunteerism means demanding something of yourself even if others do not demand anything of us (words of John Paul II),” and “By doing good to others, we do it to Christ.”

In the last part of the survey, the respondents named the traits that a volunteer should have. This was an open-ended question. Among the named traits, openness was mentioned the most often and appeared in 65% of the questionnaires. According to the respondents, communicativeness and the ability to make friends easily were equally important (51%). Additionally, the respondents emphasized the need for displaying empathy and patience, and good organization of one’s time.

## Discussion

In light of the research by Omoto and Snyder (1995), other-oriented motives are the primary factors affecting the decision to start volunteering. In turn, the self-oriented motives, connected mostly with self-development and building self-esteem, are the predictors of remaining a volunteer for a longer period of time. The results of the study presented in this paper also show that the other-oriented motives, referred to as the altruistic exogenous motives (Karyłowski 1982), affected the decision to become a volunteer during WYD. Only 10% of the respondents were volunteers for the first time. As much, 90% of the respondents were volunteers with prior experience in helping, and 32% of these volunteers helped others regularly. The volunteers considered “doing good for others” as the main rewarding factor in volunteering; these motives can also be classified as exogenous. However, the respondents most often stated that they would encourage others to become volunteers through the endogenous motives, i.e., by referring to the opportunity of gaining internal rewards in the form of a better self-esteem. The study by Houle et al. (2005) shows that potential volunteers most often do not choose the forms of help randomly, but instead try to give it sufficient thought and follow their internal motives. The people are aware that they prefer a given type of volunteerism because it corresponds with their personal needs and the aims they want to achieve. The results of this study are consistent with this assumption, i.e., volunteers indicate particular groups of people that they help, most often persons with disabilities. Experience in working with such persons motivated the volunteers to offer help to the persons with disabilities during World Youth Day. Apart from altruism, there are also other factors that motivate people to undertake pro-social activities, including volunteerism (Diacon 2014). Researchers indicate that altruism is the base on which human relations are built (Ma 2017; Elfers and Hlava 2016). One of the factors for undertaking volunteerism comprises social motives, understood as the desire to develop relations with others. According to the study conducted by the Klon/Jawor Association in Poland in 2013 on a representative sample, 77% of the surveyed Poles are of the opinion that the benefit of volunteerism is the possibility of being among people and establishing contacts, pleasure, satisfaction, a sense of accomplishment, and gaining the friendship or respect of others (Adamiak 2014). This reasoning can be associated with the social motives. In the above-mentioned study by the Klon/Jawor Association, they play a significant role. These results are not reflected in the research on volunteers assisting persons with disabilities, where the altruistic motives lead the way. Many studies show a positive effect of religion on the increase in social involvement and volunteerism (Sinha et al. 2007; Smith 2005). Religion is a strong correlate of a majority of forms of social involvement and a strong predictor of volunteerism and philanthropy (Putnam 2000). According to Priyanka and Smita, religion is closely related to altruistic activities (Priyanka and Smita 2018). Ninety-eight percent of respondents declared that they were believers and identified themselves with Catholicism. In the obtained data, religious motivations for becoming a volunteer in assisting persons with disabilities were, except for a few cases, not expressed directly.

The authors realize that the presented study has its strengths and limitation.

The strength is that the research focused on the important and current issues of interpersonal relationships and unselfish help. The study results can be important for organizations that want to find volunteers and know more about their motivation.

A limitation to this study is the fact that all participants were from one organization and from one country—Poland. Volunteers cooperating with other organizations or other countries are not included.

## Conclusions

1. The majority of volunteers from the KLIKA Association who supported persons with disabilities during WYD had experience with volunteerism.
2. Participants were driven by altruistic motives, the aim of which was to improve the situation of the persons with disabilities and enable them to participate in WYD.
3. The rewarding factor for them was, first and foremost, doing good.
4. An interesting pattern can be observed: When describing their motivations, the respondents referred to endogenous motives, and when writing how they would encourage others to become a volunteer, they referred to exogenous motives, indicating the benefits that volunteerism brings to volunteers themselves.

## References

[CR1] Adamiak, P. (2014). *Zaangażowanie społeczne Polek i Polaków. Wolontariat, filantropia, 1% i wizerunek organizacji pozarządowych* [Social involvement of Polish men and women. Volunteerism, philanthropy, the 1%, and the image of non-governmental organizations] [PDF document]. Retrieved from http://bibliotekawolontariatu.pl/wp-content/uploads/RAPORT_klon_zaangazowanie_spoleczne_2013.pdf.

[CR2] Cnaan RA, Park S (2015). Civic participation is multifaceted: Towards a pluralization of involvement. Voluntaristics Review.

[CR3] Diacon PE (2014). Pro-social behaviours: Between altruism and self-interest. Acta Universitatis Danubius.

[CR4] Elfers, J., & Hlava, P. (2016). The gratitude of caring: Altruism and empathy. In: *The spectrum of gratitude experience*. Cham: Palgrave Macmillan.

[CR5] Finkelstien MA (2009). Intrinsic versus extrinsic motivational orientations and the volunteer process. Personality and Individual Differences.

[CR6] Houle BJ, Sagarin BJ, Kaplan MF (2005). A functional approach to volunteerism: Do volunteer motives predict task preference?. Basic and Applied Social Psychology.

[CR7] Karyłowski J (1982). O dwóch typach altruizmu [On two types of altruism].

[CR8] Ma HK (2017). The development of altruism with special reference to human relationships: A 10-stage theory. Frontiers in Public Health.

[CR9] Monga M (2006). Measuring motivation to volunteer for special events. Event Management.

[CR10] Mowen JC, Sujan H (2005). Volunteer behavior: A hierarchical model approach for investigating its trait and functional motive antecedents. Journal of Consumer Psychology.

[CR11] Omoto AM, Snyder M (1995). Sustained helping without obligation: Motivation, longevity of service, and perceived attitude change among AIDS volunteers. Journal of Personality and Social Psychology.

[CR12] Penner LA, Finkelstein MA (1998). Dispositional and structural determinants of volunteerism. Journal of Personality and Social Psychology.

[CR13] Priyanka S, Smita P (2018). Life satisfaction and altruism among religious leaders. The International Journal of Indian Psychology.

[CR14] Putnam R (2000). Bowling alone: The collapse and revival of American community.

[CR15] *Regulamin Wolontariatu Światowych Dni Młodzieży Kraków* [Volunteering Regulations of World Youth Day Krakow 2016]. (2016). Retrieved from http://www.krakow2016.com/regulamin-wolontariatu.

[CR16] Sinha JW, Cnaan RA, Gelles RJ (2007). Adolescent risk behaviors and religion: Findings from a national study. Journal of Adolescence.

[CR17] Smith C (2005). Soul searching: The religious and spiritual lives of American teenagers.

[CR18] Smith DH, Stebbins RA, Grotz J (2016). Palgrave handbook of volunteering, civic participation, and nonprofit associations.

